# PL-PatchSurfer: A Novel Molecular Local Surface-Based Method for Exploring Protein-Ligand Interactions

**DOI:** 10.3390/ijms150915122

**Published:** 2014-08-27

**Authors:** Bingjie Hu, Xiaolei Zhu, Lyman Monroe, Mark G. Bures, Daisuke Kihara

**Affiliations:** 1 Department of Biological Sciences, Purdue University, West Lafayette, IN 47907, USA; E-Mails: hub@purdue.edu (B.H.); zhu313@purdue.edu (X.Z.); lmonroe@purdue.edu (L.M.); 2 Department of Computer Science, Purdue University, West Lafayette, IN 47907, USA; 3 Discovery Chemistry Research and Technologies, Eli Lilly and Company, Indianapolis, IN 46285, USA; E-Mail: bures_mark@lilly.com

**Keywords:** PL-PatchSurfer, surface patch, ligand prediction, virtual screening, 3D Zernike descriptor

## Abstract

Structure-based computational methods have been widely used in exploring protein-ligand interactions, including predicting the binding ligands of a given protein based on their structural complementarity. Compared to other protein and ligand representations, the advantages of a surface representation include reduced sensitivity to subtle changes in the pocket and ligand conformation and fast search speed. Here we developed a novel method named PL-PatchSurfer (Protein-Ligand PatchSurfer). PL-PatchSurfer represents the protein binding pocket and the ligand molecular surface as a combination of segmented surface patches. Each patch is characterized by its geometrical shape and the electrostatic potential, which are represented using the 3D Zernike descriptor (3DZD). We first tested PL-PatchSurfer on binding ligand prediction and found it outperformed the pocket-similarity based ligand prediction program. We then optimized the search algorithm of PL-PatchSurfer using the PDBbind dataset. Finally, we explored the utility of applying PL-PatchSurfer to a larger and more diverse dataset and showed that PL-PatchSurfer was able to provide a high early enrichment for most of the targets. To the best of our knowledge, PL-PatchSurfer is the first surface patch-based method that treats ligand complementarity at protein binding sites. We believe that using a surface patch approach to better understand protein-ligand interactions has the potential to significantly enhance the design of new ligands for a wide array of drug-targets.

## 1. Introduction

Interactions with small ligand molecules are essential aspects of proteins. Thus, prediction of binding ligands for a protein provides important clues of biological functions of proteins. Since close to 4000 protein tertiary structures have been solved of which function remained unknown [1], there is an urgent need for developing computational methods for structure-based function prediction. Computational prediction can help building hypothesis of protein functions that can be later tested by experiments. Binding ligands for proteins can be in principle predicted by identifying similar known binding pockets from known protein structures. There are several strategies proposed to predict binding ligands by pocket comparison in the past [2,3,4,5,6,7,8,9]. For instance, Hoffmann *et al.* measured the pockets similarity based on the alignment of protein pocket using convolution kernel between clouds of atoms in 3D space [2]. Catalytic Site Atlas [10] and AFT [11] compare a few functional residues in binding pockets and quantify the pocket similarity with the root mean square deviation (RMSD) of the residues.

Naturally, protein function prediction methods can be extended to identify chemical compounds that bind to a target protein as a part of drug design. In the drug discovery field, there are two major categories of computational methods for binding ligand prediction: ligand-based methods and structure-based methods. The ligand-based methods derive critical chemical features from a compound or set of compounds that are known to bind to a target and use these features to search for compounds with similar properties in a virtual compound library. This can be done by a variety of methods, including similarity and substructure searching [12,13,14,15], 3D shape matching [16,17], and searching with Quantitative Structure-Activity Relationship (QSAR) models [18,19,20,21]. The advantage of such methods is that no target information is required. However, a major drawback of the ligand-based approaches is its dependency on the chemical features present in the known actives. Physico-chemical features that are absent in the set of active compounds used to derive the model are often neglected. Therefore, active compounds with novel scaffolds are rarely, if ever, recognized during the screening process.

Alternatively, when the structure of the target protein is known, structure-based methods can be performed. Structure-based methods do not require *a* *priori* knowledge of active ligands; therefore the models are not biased by the chemical space of previously identified actives. One of the most widely used structure-based tools is molecular docking. The aims of docking are to predict the correct binding pose of a small molecule in the target protein’s binding site and to provide an estimate of the affinity of the small molecule. Many docking programs have been developed in the past decades and have been successfully applied in virtual screening studies [22,23]. In the molecular docking programs, the protein and the ligand are described by one of the three representations: grid, atomic, and surface [24]. The grid representation, such as GRID [25], stores the receptor’s energy contribution on the grid points to accelerate the scoring of the ligand poses in the initial search algorithms. Therefore, it is widely used in various docking programs in the early stage of the ligand pose selection. The atomic representation is generally used in the final scoring of the binding poses in combination with an atom-based potential energy function [24], as used in AutoDock [26,27], Glide [28], DOCK [29], PharmDock [30], and many other docking programs [24]. The surface based representation, on the other hand, is typically used in protein–protein docking [31,32,33], such as LZerD [34] and ZDOCK [33].

In our efforts for predicting the functions of proteins, we have developed an alignment free surface-based pocket comparison program named PatchSurfer [8,35]. PatchSurfer represents a binding pocket as a combination of segmented surface patches, each of which is characterized by its geometrical shape, the electrostatic potential, the hydrophobicity, and the concaveness. The shape and the three physicochemical properties of surface patches are represented using the 3D Zernike descriptor (3DZD), which is a series expansion of mathematical 3D function [36,37]. Given a query pocket in a protein, PatchSurfer searches a database of known pockets and finds similar ones to the query based on the surface-patch similarity. PatchSurfer was benchmarked on three different datasets and has shown superior performance to existing methods [8].

PL-PatchSurfer is being developed to explore the utility of including ligand patch surfaces in our existing PatchSurfer methodology. PL-PatchSurfer represents both a protein binding pocket and a ligand molecule by their surface properties and identifies the optimal complementarity between the pocket and the ligand surface. The advantages of the surface representation are that it is less sensitive to subtle changes on the pocket and ligand conformation and the search speed can be quite fast. Each surface patch characterizes geometrical and physicochemical properties of a protein pocket and ligand on a continuous surface. We first tested the PL-PatchSurfer on the binding ligand prediction problem using a dataset with 146 protein structures binding with 12 different ligand types and studied the influence of the ligand conformations on the performance of PL-PatchSurfer. We then evaluated and optimized the performance of PL-PatchSurfer in identifying the native contacts on a large set of known protein-ligand complex structures from the PDBbind database [38,39]. Finally, we tested PL-PatchSurfer on the directory of useful decoys (DUD) dataset to examine how it performs in virtual screening on a large and structurally diverse dataset. To the best of our knowledge, PL-PatchSurfer is the first surface patch-based method being developed that utilizes descriptors derived from the surface properties of ligands. The performance of PL-PatchSurfer when compared to our previous benchmarks using a pocket-based approach sheds further light on the utility of surface-based methods. In the Conclusions, we summarize characteristic performance of PL-PatchSurfer and discuss usefulness of the new approach.

## 2. Results and Discussion

### 2.1. Binding Ligand Prediction on Huang Data Set

PatchSurfer was originally developed for predicting the functions of unknown proteins based on ligand binding site similarities to known proteins. In our previous study [8], we have used PatchSurfer to predict the binding ligands of the proteins in the Huang data set [40] (Table 1) based on the principle that structurally similar binding pockets would bind similar ligands. PL-PatchSurfer takes a complementary approach to Patch-Surfer in that it predicts the binding ligand of a given protein based on the molecular surface complementarity between the ligand and the protein pocket. As a comparison with PatchSurfer, we first tested the performance of PL-PatchSurfer on the Huang dataset. Table 1 summarizes the number of binding pockets of twelve different ligand types in the dataset as well as the average number of surface patches that represent the pockets and the ligand molecules. The number of pocket patches and ligand patches correlates well with a correlation coefficient of 0.953. For each ligand, a maximum of 20 ligand conformations were generated using the Omega program from OpenEye [17,18,19]. Dependent on the rigidity of the ligands, some ligands have no more than five ligand conformations.

**Table 1:** Huang data set.

Ligand Name	# Pockets	# Omega Conformers	Avg # Pocket Patches	Avg # Ligand Patches
**AND**	12	20	22.3	18.1
**BTN**	8	20	18.6	17.7
**F6P**	10	20	19.8	16.5
**FUC**	8	2	8.6	11.5
**GAL**	32	3	13.9	12.7
**GUN**	11	1	14.6	10.0
**MAN**	15	6	9.3	11.5
**MMA**	8	10	13.0	13.4
**PIM**	5	2	14.0	12.0
**PLM**	24	20	28.3	24.1
**RTL**	5	20	31.2	25.9
**UMP**	8	20	22.5	19.2
**Total**	146	144	-	-

AND: adenosine; BTN: biotin; F6P: fructose 6-phosphate; FUC: fucose; GAL: galactose; GUN: guanine; MAN: mannose; MMA: O1-methyl mannose; PIM: 2-phenylimidazole; PLM: palmitic acid; RTL: retinol; UMP: 20-deoxyuridine 5-monophosphate; #: Number.

The procedure for testing the performance of PL-PatchSurfer on the Huang data set was as follows: Each ligand binding pocket was selected as a query, which was compared with ligands in the dataset and the similarity score between the pocket and each ligand was computed using the so-called *Totalscore_PS_* (Equation (13); see the Experimental Design section). The *Totalscore_PS_* quantifies the similarity of a pocket and a ligand molecule by considering local surface similarity, relative positions of corresponding surface patches on the pocket/ligand, and the size of the pocket/ligand using corresponding patch pairs identified by a distance score of patches (Equation (12)). The ligands were sorted by the *Totalscore_PS_*, which were used to finally predict binding ligands for the query pocket by the PocketScore (Equation (16)). 

**Figure 1:**
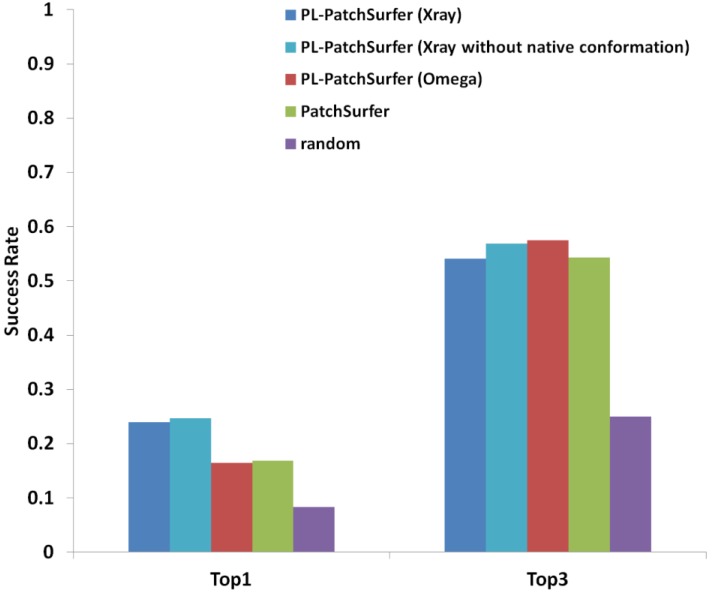
Performance of PL-PatchSurfer and PatchSurfer on the Huang data set. For PL-PatchSurfer, three tests were performed: (i) X-ray: the bound ligand conformations of all the tested proteins are extracted to form the “X-ray ligand conformation database”; (ii) X-ray without native conformation: the native ligand conformation of the query pocket was removed from the “X-ray ligand conformation database”; and (iii) Omega: A maximum of 20 ligand conformations with the lowest internal energies are computationally generated by OpenEye Omega. The data for PatchSurfer was extracted from a previous publication [8].

Two tests were performed with PL-PatchSurfer. First, we used the bound ligand conformation from the X-ray crystal structure. For a query pocket, the bound conformation for the pocket itself was either included or excluded from the ligand dataset. The fraction of query pockets whose bound ligand type was correctly predicted at the highest score or within the top-3 highest scoring ligands were reported in Figure 1. Compared to the results from pocket-pocket comparisons using PatchSurfer, PL-PatchSurfer performs 7.1% better in ranking the correct binding ligand at the top-1 position. When within top-3 positions were considered, PL-PatchSurfer still performed slightly better than PatchSurfer. Interestingly, excluding the native ligand conformation of the query pocket did not change the result much; actually it even showed slight improvement in the success rate.

It is remarkable that the PL-PatchSurfer showed a higher success rate than PatchSurfer, because PatchSurfer has been extensively compared with existing methods in our previous works. It was demonstrated in our previous paper that the patch-representation for pockets used in PatchSurfer was effective in achieving a higher accuracy than PocketSurfer [36], which represents pockets as a rigid pocket with a single surface descriptor [8]. It was also shown that the 3D Zernike descriptor, a mathematical surface representation used in PatchSurfer (see Experimental Design for more about the 3D Zernike Descriptor), had a higher accuracy than similar mathematical surface representations, spherical harmonics [41] 2D Zernike descriptor, pseudo-Zernike descriptors, and Legendre moments [8]. Moreover, Patch-Surfer also showed better prediction performance than four existing methods, eFseek [42], SiteBase, PROSURFER, and XBSite2F [37].

In a ligand virtual screening experiment, a bound ligand conformation for a target pocket is not always available. Furthermore, the ligand conformation with the lowest internal energy is not necessarily the bound conformation for a target-binding site. Therefore, a set of ligand conformations are usually pre-generated before performing virtual screening or are generated on-the-fly during a screening process. In the second test we used computer-generated ligand conformations using the Omega program from OpenEye [43,44,45] (Table 1). As shown in Figure 1, the top-1 success rate of PL-PatchSurfer using Omega-generated conformers was lower than the top-1 success rate using X-ray ligand conformations. However, interestingly, the top-3 success rate using Omega-generated ligand conformers was slightly higher than the top-3 success rate using the X-ray ligand conformations. In summary, the results on the Huang dataset show that the ligand-based patch method implemented in PL-PatchSurfer gives significant higher accuracy in the top-1 success rate than PatchSurfer, which indicates that correct ligands are recognized in a more specific fashion by PL-PatchSurfer than PatchSurfer. In the top-3 success rate, improvement by PL-PathSurfer is marginal but still showed better results than PatchSurfer.

### 2.2. Optimization of the Search Algorithm on PDBbind Dataset

In the work described for the Huang dataset, we used PL-PatchSurfer with parameters that were optimized for pocket-to-pocket comparison in PatchSurfer (Equations (12) and (13) in the Methods section). In this section, we optimize the parameters in Equation (4) that determine contributions of different terms in the overall ligand-pocket matching score. A key step in PL-PatchSurfer is identification of corresponding surface patch pairs from a pocket and from a ligand by minimizing a distance score, which is a linear combination of the differences in 3DZD, the relative geodesic position, and the relative geodesic distance (Equation (4)). We optimized the weights in the distance score (Equation (4)) using the PDBbind core set [38,39].

**Figure 2:**
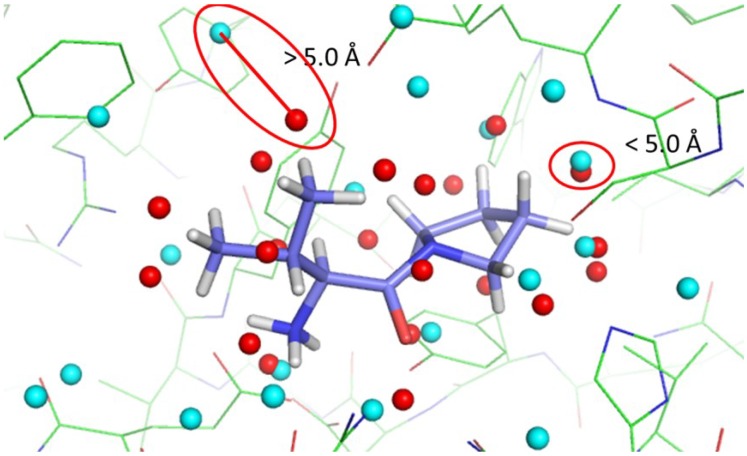
Definition of the success rate in determining correctly matched patches. For each protein–ligand complex structure, protein patches (cyan spheres) and ligand patches (red spheres) are generated. Using PL-PatchSurfer, all potential matching pairs were identified (examples of two potential matching pairs are shown in the red ellipses). Successful matches are defined as those where the distance between a paired ligand patch and a protein patch is within a cutoff distance.

Ideally, a ligand patch identified as a match to a protein pocket patch should localize in the vicinity of the given protein patch in the ligand-bound structure of the protein to form inter-molecular interactions. To evaluate the performance of PL-PatchSurfer in identifying matching patches, we computed the match success rate on known protein–ligand complex structures from the PDBbind core set. If the distance between the centers of the identified matching patches is within a cutoff distance, we considered that the matching pair was correctly identified and counted it as a success (Figure 2). As a cutoff distance, primarily we used 5.0 Å but also tested 3.0, 4.0, and 6.0 Å. The match success rate is the number of correct contacting patches identified by PL-PatchSurfer divided by the actual number of correct contact pairs between the protein and the ligand for each complex structure (see Experimental Design for details).

**Figure 3:**
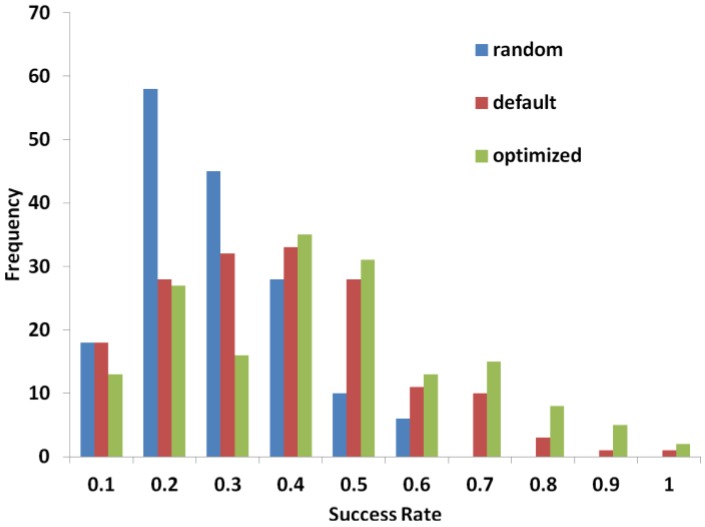
Distribution of patch matching success rate. Frequency on the *y*-axis counts the number of individual protein–ligand complexes of a success rate. The distance threshold was set to 5.0 Å.

We first tested PL-PatchSurfer with the default weights that were optimized for PatchSurfer in the pocket–pocket comparison [8], which showed a success rate of 33.1% for the 5.0 Å cutoff distance (Table 2). We then optimized the weights following the approach described in the Method section. After the optimization process, the success rate increased to 39.1% with an average of 10 correct contacting patch pairs identified for each protein-ligand complex. Table 2 also shows the optimization is effective over a range of distance thresholds. The distribution of the success rate for individual protein–ligand complexes is plotted in Figure 3. It is clearly observed that the distribution of the match success rate was improved by the optimization.

**Table 2:** Coefficients and the success rate of different distance score functions.

Setting	3DZD Difference	Geodesic Distribution	Geodesic Distance	Success Rate			
				3.0 Å	4.0 Å	5.0 Å	6.0 Å
**Default ^1^**	0.32	0.48	0.2	12.3%	21.6%	33.1%	44.0%
**Optimized **	0.35	0.15	0.5	15.2%	26.2%	39.1%	51.1%
**Random ^2^**	-	-	-	7.6%	13.4%	23.7%	35.8%

^1^ The coefficients are optimized for PatchSurfer in pocket–pocket comparison studies; ^2^ Matching patch pairs were randomly generated in the search process.

### 2.3. Performance of PL-PatchSurfer on Directory of Useful Decoys (DUD) Data Set

In this next experiment, we explored the utility of using PL-PatchSurfer in virtual screening exercise on the DUD dataset, which is large and contains diverse active and compound data. Knowing that PL-PatchSurfer performs well in binding ligand prediction, we wanted to investigate how it performs in virtual screening. A training process was carried out to identify the best parameters for the scoring function (Equation (8)) used in ranking the ligand. We split the protein targets into the training and testing set randomly, with 12 targets for training and 13 for testing. Optimization of the training set results lead to a parameter set of 0.8, 0.0, 0.1 as weights for *Totalscore_PL_* (Equation (8)). A cross-validation switching the training and the testing sets yielded a parameter set of 1.0, 0.0, 0.6, which is similar to the ones initially obtained. Interestingly, both parameter sets suggest that the relative distance score, *avgGrpd* (Equation (10)) in *Totalscore_PL_* (Equation (8)) does not contribute to the discrimination between the active and the decoy ligands. However, as the relative distance term *grpd* (Equation (7)) is also used in *Distance_score_PL_* (Equation (4)), which is used for finding matching patch pairs by the auction algorithm, the relative patch distance information is indirectly used in the entire search process. The weight of 0 for the relative distance term in *Totalscore_PL_* indicates that this term does not contain useful information for distinguishing different ligands in this dataset. However, the *Totalscore_PL_* term is useful for identifying corresponding patch pairs for a given pocket–ligand pair by *Distance_score_PL_*. Our optimization results suggest that differences in the 3DZD fingerprints are the major discriminator in ranking the active and decoy ligands.

**Figure 4:**
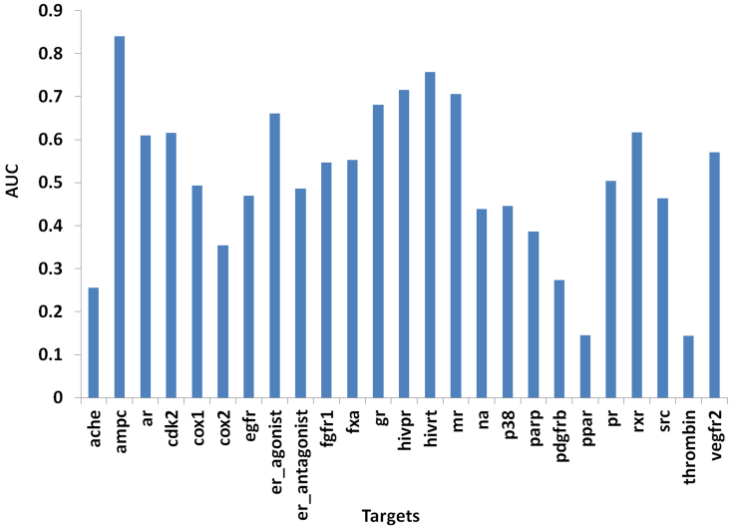
Area-under-the-curve (AUC) values for individual protein target. Enzyme abbreviations: AChE, acetylcholinesterase; AmpC, AmpC β-lactamase; AR, androgen receptor; CDK2, cyclindependent kinase 2; COX-1, cyclooxygenase-1; COX-2, cyclooxygenase-2; DHFR, dihydrofolate reductase; EGFr, epidermal growth factor receptor; ER, estrogen receptor; FGFr1, fibroblast growth factor receptor kinase; FXa, factor Xa; GR, glucocorticoid receptor; HIVPR, HIV protease; HIVRT, HIV reverse transcriptase; MR, mineralocorticoid receptor; NA, neuraminidase; P38 MAP, P38 mitogen activated protein; PARP, poly(ADP-ribose) polymerase; PDGFrb, platelet derived growth factor receptor kinase; PPARg, peroxisome proliferator activated receptor γ; PR, progesterone receptor; RXR, retinoic X receptor α; SRC, tyrosine kinase SRC; TK, thymidine kinase; VEGFr2, vascular endothelial growth factor receptor.

**Figure 5:**
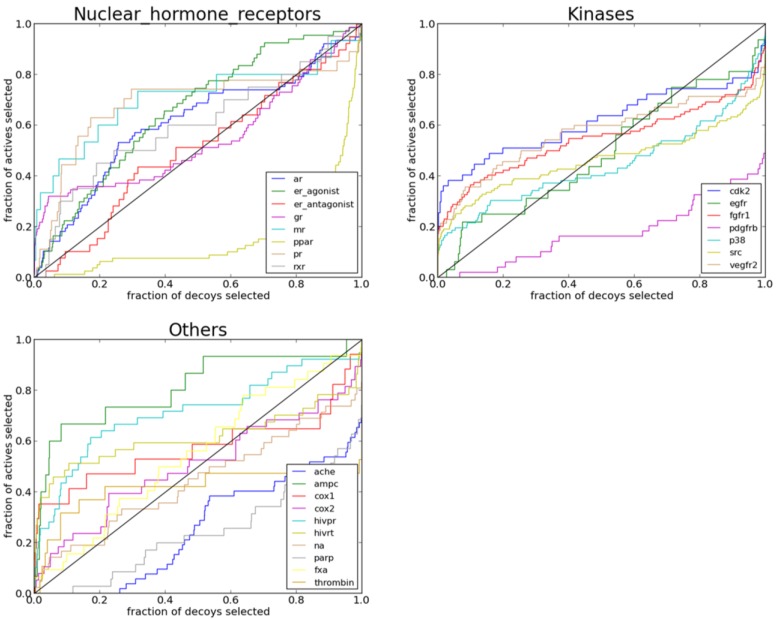
ROC plots for individual targets in the directory of useful decoys (DUD) dataset.

The area-under-the-curve (AUC) of the Receiver Operating Characteristic (ROC) plot for each protein target when its used in the testing set is shown in Figure 4. Overall, PL-PatchSurfer is able to provide a better than random (AUC of 0.5) AUC value for 12 out of the 25 protein targets. Notably however, PL-PatchSurfer was able to achieve an AUC over 0.70 for four protein targets: ampc, hivpr, hivrt, and mr. It is also interesting to note that ROC plots for individual targets in Figure 5 show that significantly more actives than decoys were selected in early ranks for 20 out of 25 targets including the targets that have AUC values below 0.5 (P38, SRC, cyclooxygenase-1 (COX1) and cyclooxygenase-2 (COX2)). This is an important indicator that PL-PatchSurfer can be used to effectively prioritize compounds for experimental testing.

To further understand characteristic performance of PL-PatchSurfer, we analyzed the results for two targets, ampc, and fgfr1, where PL-PatchSurfer generated significantly greater enrichment factors in a virtual screening exercise when compared to PharmDock [30], as shown in Table 3. We have chosen PharmDock because its performance was extensively compared with other existing program on the virtual screening performance over the DUD dataset by Hu and Lill [30]. PharmDock has been compared with six docking programs, DOCK [29], FlexX [46], Glide [28], ICM [47], Surflex [48], and PhDock [49]. Overall, PharmDock has been shown to have a better performance than DOCK and PhDocK and comparable performance with ICM and FlexX. In Table 3, we compared enrichment factors of PL-PatchSurfer and PharmDock on the 25 targets at 1%, 10%, and 20% levels. An enrichment factor at *X*% indicates how well hits within X% are dominated by actives; concretely, the percentage of actives within top *X*% hits is normalized by the overall fraction of actives in the compound dataset. At the EF1% and and EF10%, PL-PatchSurfer showed larger average enrichment factors than PharmDock: At 1%, the enrichment of PL-PatchSurfer and PharmDock was 8.63 and 6.87, while at 10% it was 2.48 and 2.23 for PL-PatchSurfer and PharmDock, respectively. PL-PatchSurfer showed a slightly smaller enrichment of 1.68 at 20%, where PharmDock had 1.72. Thus, on average PL-PatchSurfer was superior to PharmDock in early enrichment, which is practically one of the most important characters in virtual screening.

**Table 3:** Enrichment factors for PL-PatchSurfer and PharmDock.

Protein	PL-PatchSurfer			PharmDock		
	EF1%	EF10%	EF20%	EF1%	EF10%	EF20%
**AChE**	0.00	0.00	0.00	0.00	0.19	0.14
**AmpC**	11.27	6.63	3.65	0.00	0.48	0.95
**AR**	0.00	1.81	1.62	12.16	2.43	2.09
**CDK2**	25.26	3.81	2.34	2.00	2.40	2.20
**COX1**	23.41	3.51	2.34	4.00	0.80	0.40
**COX2**	2.56	1.57	1.05	4.60	1.67	1.05
**EGFr**	0.00	2.18	1.24	2.25	2.16	1.94
**ER_agonist**	0.00	2.23	1.64	2.99	5.37	3.21
**ER_antagonist**	0.00	0.51	0.64	12.82	3.59	2.69
**FGFr1**	18.89	3.45	1.97	0.85	0.17	0.55
**FXa**	5.86	1.25	1.40	3.52	2.46	2.08
**GR**	14.89	3.33	1.79	11.54	1.92	1.35
**HIVPR**	15.18	5.09	3.20	24.53	7.17	4.06
**HIVRT**	27.86	5.10	2.83	7.50	1.75	1.75
**MR**	17.97	4.66	2.66	26.67	6.00	3.67
**NA**	2.37	1.66	0.95	4.08	1.84	1.12
**P38**	12.56	2.15	1.52	1.95	1.60	1.54
**PARP**	0.00	0.00	0.14	36.36	4.85	3.03
**PDGFrb**	0.00	0.20	0.41	0.00	0.38	0.45
**PPARg**	0.00	0.00	0.06	0.00	0.00	0.43
**PR**	3.60	2.20	2.77	3.70	2.22	1.48
**RXR**	0.00	2.96	2.23	0.00	1.00	2.25
**SRC**	15.99	2.90	1.87	0.65	2.13	1.84
**thrombin**	0.00	2.08	1.57	1.54	0.92	1.15
**VEGFr2**	17.96	2.71	2.00	8.11	2.16	1.69

EF1%: enrichment factors at 1% ranked decoys; EF10%: enrichment factors at 10% ranked decoys; EF20%: enrichment factors at 20% ranked decoys.

**Figure 6:**
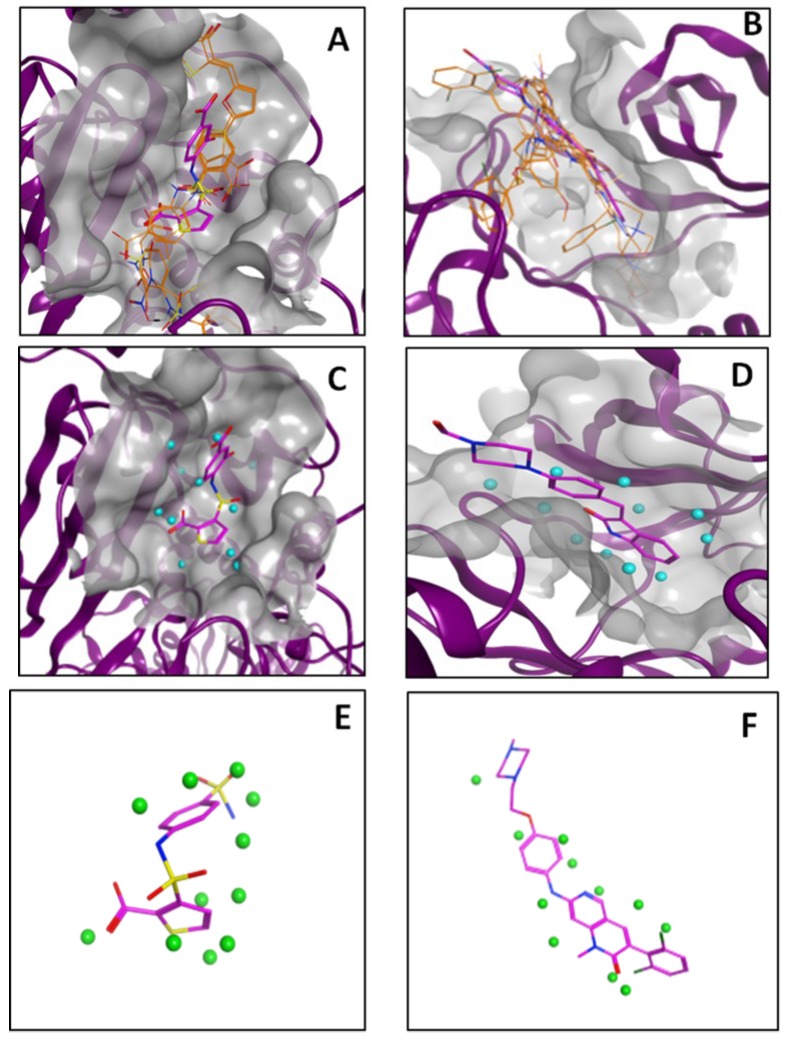
Panels (**A**) and (**B**) show that the top six PharmDock-generated poses (orange) are not similar to the ligand binding orientation found in the crystal structures of AmpC (panel **A**) and FGFr1 (panel **B**); The cyan spheres depicted in Panels (**C**) and (**D**) represent binding sites patches, generated by PL-PatchSurfer, that correspond to productive protein–ligand interactions observed in crystal structures of AmpC (PDB ID: 1XJG) and FGFr1 (PDB ID: 1AGW), respectively; The green spheres highlighted in panels **E** and **F** are the ligand patches (for a related AmpC-active and FGFr1-active ligand, respectively) that are matched by PL-PatchSurfer to the binding site patches, showing good coverage of the protein-ligand surface interaction space.

We also took a closer look at results on two targets, ampc and fgfr1. Looking first at the PharmDock results, we observed that none of the top-scoring poses of actives predicted by PharmDock were similar to a ligand binding pose observed in the AmpC (Figure 6A) and FGFr1 (Figure 6B) crystal structures represented in the DUD set. For AmpC (Figure 6A), the distance from the centroid of the bound ligand to the centroids for the poses of the six active compounds ranged from 4.7 to 8.7 Å, while it ranged from 3.2 to 5.4 Å for FGFr1. Therefore, given this observation, the low enrichment factors generated by PharmDock for these two targets is perhaps not surprising. Turning to the PL-PatchSurfer results, we found good correspondence between the matched protein-binding site and ligand patches. This observation is illustrated in Figure 6, where panels C and D show the generated pocket patches (cyan spheres) that represent productive protein-ligand binding interactions present in the ampc and FGFr1 crystal structures respectively; while panels E and F show ligand patches (green spheres) that are matched (by PL-PatchSurfer) to the highlighted binding sites patches in ampc and FGFr1, respectively. We compared the positions of matched patches since PL-PatchSurfer does not explicitly provide orientations of bound compounds. These sets of matched patches shows that there is good coverage of the binding regions that are making productive interactions observed in the ampc and FGFr1 crystal structures, shedding some light on scenarios where PL-PatchSurfer is able to generate good enrichment factors in virtual screening exercises.

AChE, PPAR, COX-2, PDGFrb, and thrombin appear to be difficult targets for PL-PatchSurfer. For these difficult targets, we performed a target-specific training process on a randomly select subset of active and decoy ligands from each target (see Experimental Design Section for detail). The resulting parameters and AUC values for these targets are shown in Table 4. Significant improvement in the AUC values was observed after this target-specific training. However, the parameter sets obtained were very different from the ones obtained in Figure 4 with the weight of 0 for the 3DZD similarity. This may be partly due to a limited number of physicochemical features considered in the current implementation of PL-PatchSurfer. The two features currently considered, the surface shape and the electrostatic potential, may not be sufficient in discriminating binding ligands for targets where other types of intermolecular interactions, such as hydrogen bonds, hydrophobic, and aromatic interactions, play critical roles. For PPAR and PDGFrb, both targets were found to be difficult for the widely used docking programs, such as DOCK and PhDOCK [50,51].

**Table 4:** Target-specific training results for the five difficult targets.

Protein	3DZD	Relative Distance	Pocket Size	AUC	AUC before Training
**AChE**	0	0.3	0.9	0.60	0.26
**PPAR**	0	1.0	0	0.68	0.15
**COX-2**	0	0	1.0	0.56	0.35
**PDGFrb**	0	1.0	1.0	0.42	0.27
**Thrombin**	0	0	1.0	0.50	0.14

### 2.4. Comparison of Computational Time

At the end of the Result section, we compared the computational time of PL-PatchSurfer in comparison with PharmDock, AutoDock, and Glide (Table 5). For this test, we used ten targets in the DUD dataset. PL-PatchSurfer took up to about a second to search one ligand against a given target and clearly the fastest among these four methods. PL-PatchSurfer is on average 40 to 500 times faster (average 266.4 times) than PharmDock, on average 30.2, 80.4 times faster than AutoDock and Glide, respectively.

Times are in seconds. Jobs were run on a Linux machine with Intel Core i7-3820 CPU, 3.60GHz, with 65 GB RAM. The times counted are only for searching steps excluding file preparation steps. The times for AutoDock and Glide were taken from log files output by the programs. The rigid docking mode was used for AutoDock and Glide.

**Table 5:** Computational time of four methods.

System	PharmDock	PL-PatchSurfer	AutoDock	Glide
**10gs**	585.5	1.2	43	54
**1a30**	74.0	0.5	30	48
**1bcu**	15.0	0.4	5	22
**1gpk**	22.0	0.5	7	33
**1h23**	988.9	0.8	57	48
**1lol**	64.6	0.7	15	129
**1loq**	42.6	0.7	12	120
**1mq6**	348.9	0.7	34	28
**1n2v**	15.6	0.4	6	25
**1q8t**	25.9	1.3	8	31

## 3. Experimental Design

**Figure 7:**
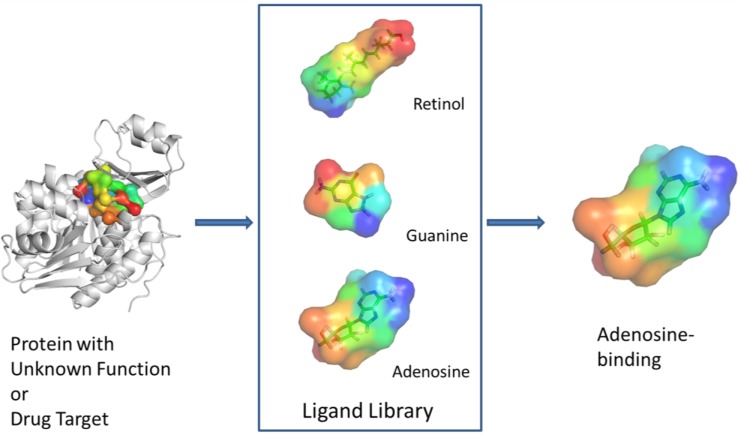
Overall scheme of PL-PatchSurfer.

In this section we describe procedures and datasets used in this work. The overall scheme of PL-PatchSurfer is depicted in Figure 7. Given a protein with unknown function or a protein target of interest, its ligand-binding pocket will be extracted. The surface of the binding pocket will be represented by a set of segmented surface patches, each of which is described by its surface shape and electrostatics potential. The pocket will then be used to search against a ligand library, where each ligand is also represented by a set of surface patches. The ligands will be ranked based on their surface complementarity with the protein-binding pocket and their molecular size to suggest the best binding ligands for the target protein. The details of each step will be described below.

### 3.1. Definition of the Protein Pocket Surface

The surface of a protein is computed with the Adaptive Poisson–Boltzmann Solver (APBS) program [52] which defines the surface as the boundaries of solvent accessible and solvent excluded regions. Surface shape information is stored in a 3D grid where grid points that overlap with the protein surface are specified. The electrostatic potential of the protein is also computed using the APBS program and the energy values are assigned onto each grid point. The center of the protein pocket is defined by the center of mass of its known binding ligands. The pocket surface is then defined as surface points that are encountered by rays cast from the center of the protein pocket. The detailed description of the ray-casting method can be found in our previous publication [36].

### 3.2. Generation of Ligand Conformations and Computation of the Surface Properties

To account for the ligand flexibility, multiple ligand conformations were generated using OpenEye Omega (OpenEye Scientific Software Inc. Santa Fe, NM, USA [43,44,45]. For each ligand, a maximum of 20 conformations are generated with the calculated internal energy no more than 15 kcal/mol above the energy of the ligand conformation with the lowest internal energy. Duplicate conformers are removed using a 0.5 Å root-mean-square deviation (RMSD) cutoff for ligands with zero to five rotatable bonds, a 0.8 Å cutoff for ligands with six to ten rotatable bonds, and a 1.0 Å cutoff for all ligands with more than ten rotatable bonds. For each ligand conformation, the APBS program is used to compute the surface of the ligand and the electrostatic potential of the ligand on the surface. The surface shape and the electrostatic potential are also mapped onto 3D grid points for subsequent identification of the surface patches.

### 3.3. Identification of the Surface Patches

Based on the defined surface of the binding pocket or the ligand, PL-PatchSurfer identifies a group of patches covering the surface area. First, a set of seed points is selected as the centers for the patches. The seed points are iteratively selected from the surface points that are closest to protein or ligand heavy atoms within 3.5 Å to the defined surface [8]. The minimum distance between any pairs of seed points are set to be 3.0 Å in order to distribute the patches evenly over the pocket surface. Finally, a patch is defined as a connected single surface region within 5.0 Å from a center seed point.

### 3.4. Computation of the 3D Zernike Descriptors on the Surface Patches

The 3D Zernike Descriptors (3DZD) is a series expansion of a 3D function, which allows compact and rotationally invariant representation of a 3D function [53]. The detailed description of 3DZD can be found in the references [53,54] and our previous studies [8,36]. Briefly, for each identified patch that consists of connected surface points, there are two 3D functions representing the surface shape and the electrostatic distribution: *f_shape_*(*x*) and *f_elec_*(*x*). The 3D functions can be expanded into a series in terms of Zernike–Canterakis basis defined as follows:

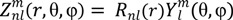
(1)
where −*l* < *m* <*l*, 0 ≤ *l* ≤ *n*, and *n-l* even. 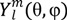 is the spherical harmonics and *R_nl_*(*r*) is the radial function constructed in a way that 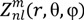 can be converted to polynomials in the Cartesian coordinates, 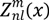. To obtain the 3DZD of *f*(*x*), first 3D Zernike moments are computed:

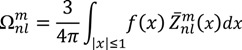
(2)


Then, the 3DZD, *F_nl_* is computed as norms of vectors *Ω_nl_*. The norm gives rotational invariance to the descriptor:

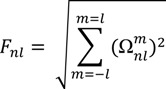
(3)


We used *n* = 15 so that the shape is represented by a vector of 72 invariant values. The electrostatic potential is represented by 144 = 72 × 2 invariants, since a 3DZD is computed each for positive electrostatic values and negative electrostatic values.

### 3.5. Procedure of Protein–Ligand Patch Comparison

The procedure of comparing the complementarity between the pocket and the ligand can be summarized in the two steps: (i) search the optimal matching patch pairs between the pocket and the ligand; and (ii) compute the distance score between the pocket and the ligand.

#### 3.5.1. Search Matching Patches between the Pocket and the Ligand

We used the auction algorithm [7,55] to search for the optimal matching patch pairs that yield the minimum distance score for the pocket and the ligand pair. The distance score between patch *a* from the pocket *A* and patch *b* from the ligand *B* is computed by:

*Distance_score_PL_*(*a, b*) = *w*_1_ × *pdist*(*a, b*) + *w*_2_ × *appd*(*a, b*) + *w*_3_ × *grpd*(*a, b*) (4)

where *pdist*(*a*, *b*) is the weighted sum of the Euclidean distances (*L*2 norm) between the 3DZDs of the surface shape and electrostatic potential:

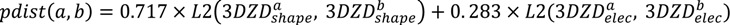
(5)


The weights, 0.717 and 0.283, are for normalizing the difference in the value distribution of the shape and the electrostatic properties and were trained from the previous studies [8].

The second term *appd*(*a, b*) compares the relative position of the patch *a* on the surface of pocket A with the patch *b* on the surface of ligand B. It is computed using the patch distribution vector which describes the approximate patch position (APP) feature for each patch. This feature approximately describes the relative position of a given patch in a pocket or a ligand, namely, if a patch is in the middle or on the edge of a pocket/ligand. To compute APP, we calculated the geodesic distance between each pair of patches. The geodesic distance is the distance between the two patch centers along the molecular surface. For each patch, its geodesic distances to the other patches were binned to render a patch distribution vector with the numbers of patches in different bins. A bin size of 1.0 Å and a total number of 40 bins were used. The *appd*(*a, b*) is then calculated by:

*appd*(*a, b*) = *L*2(*APP*_a_, *APP*_b_) (6)

The last term *grpd*(*a, b*) measures the geodesic relative position difference:

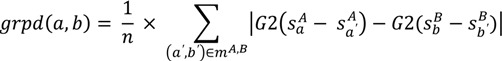
(7)
where *m^A,B^* contains a list of the identified matching patch pairs in the previous search steps of the Auction algorithm. And *n* is the number of the identified matching patch pairs in the previous steps, *i.e.*, the length of *m^A,B^*. When *n* is zero, *i.e.*, in the first search step of the Auction algorithm, this term is ignored. (*a’*, *b’*) is the matching pair belongs to *m^A,B^*. 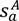 is the coordinate of the center of the patch in pocket *A* of matched pair *a*. *G*2 is the geodesic distance between the centers of the two patches.

The three terms *pdist*(*a*, *b*), *appd*(*a*, *b*), and *grpd*(*a*, *b*) are linearly combined in Equation (4). Their coefficients are trained on the PDBbind dataset as will be described below in Section 3.6.2.

#### 3.5.2. Score the Overall Fit of the Ligand into the Pocket

To measure the overall fit of the ligand *B* into the protein pocket *A*, the following scoring function was used:

*Totalscore_PL_*(*A, B*) = *w*_1_ × *avgZd*(*A, B*) + *w*_2_ × *avgGrpd*(*A, B*) + *w*_3_ × *pocketSd*(*A, B*) (8)

The first term is the average distance score between the matching patches, defined as:

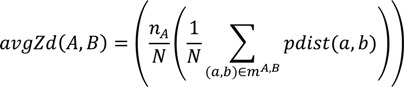
(9)
where *n_A_* is the number of patches in the protein pocket *A*. *N* is the number of matching patch pairs between pocket *A* and ligand *B*. *pdist* is the distance score of two patches as defined in Equation (5). *m^A,B^* contains the list of matched patch pairs from pockets *A* and ligand *B*.

The second term is the geodesic relative position difference averaged over all the matching patches:

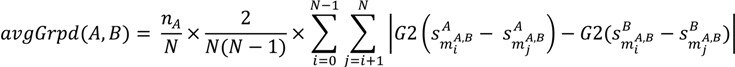
(10)
where *G*2 is the geodesic distance between the centers of the two patches.

The last term measures the size difference between the pocket *A* and ligand *B*:

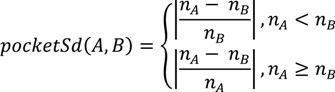
(11)
where *n_A_* is the number of patches in the protein pocket *A* and *n_B_* is the number of patches in the ligand *B*. The three terms are linearly combined in Equation (8).

### 3.6. Data Set and Evaluation Methods

#### 3.6.1. Huang Dataset

The Huang dataset [40] was originally curated for testing the ligand binding site prediction programs and was used for examining pocket retrieval performance of PatchSurfer in the previous study [8]. There are a total of 146 proteins that bind with one of the 12 ligand molecules (Table 1). The sequence identity between each pair of proteins is lower than 30%. For each protein, the ligand binding pocket was defined using the known binding ligand as described in Section 3.1. The pocket patches were then identified as described above. The average number of patches identified for each group of proteins was listed in Table 1.

We used the Huang data set to investigate whether OpenEye Omega [43,44,45] is able to produce ligand conformations that can achieve the ligand prediction results comparable with that of using the X-ray ligand conformations. For this purpose, the native ligand of each protein was extracted to form the X-ray ligand conformation set. Meanwhile, a maximum of 20 ligand conformations is generated for each ligand using OpenEye Omega with the parameters described in Section 3.2. The patches were then identified for each ligand conformation following the method described in Section 3.3 and Section 3.4. We used PL-PatchSurfer to predict the binding ligand of each protein using both X-ray and Omega-generated ligand sets.

To compare the performance of PL-PatchSurfer in predicting the binding ligand with the PatchSurfer, we used the same *Distance_score* from PatchSurfer [8] for identification of matching patches:

*Distance_score_PS_*(*a, b*) = *pdist*(*a, b*) (12)

where the *pdist* is as described in Equation (5). Thus, compared with *Distance_score_PL_*, only similarity of 3DZDs of the surface shape and the electrostatic potential was considered in *Distance_score_PS_*.

The *Totalscore* from PatchSurfer were used for scoring the ligand against the pocket:

*Totalscore_PS_*(*A, B*) = 0.06 × *avgZd*(*A, B*) + 0.14 × *rdp*(*A, B*) + 0.8 × *pocketSd*(*A, B*) (13)

where *avgZd* is as described in Equation (9). *rdp* is the relative distance between the matching patches based on the Euclidean distance:

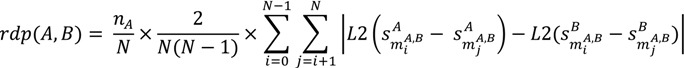
(14)
where *n_A_* is the number of patches in the protein pocket *A*. *N* is the number of matching patch pairs between pocket *A* and ligand *B*. *L*2 is the Euclidean distance between the centers of the two patches.

Finally, the pocket size is computed by:

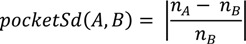
(15)
where *n_A_* is the number of patches in the protein pocket *A* and *n_B_* is the number of patches in the ligand *B*.

The final score of a ligand matching with a protein is computed by:

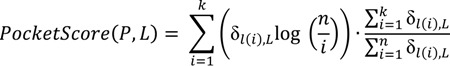
(16)
where *l*(*i*) denotes the ligand type (e.g., AMP, FAD, *etc.*) of the *i*-th ranked ligand to the query, *n* is the number of ligands in the database, and the function *δ*_*l*(*i*)*,L*_ equals to 1 if the *i*-th ranked ligand conformation is from ligand *L*, and is 0 otherwise. The first term is to consider *k* top-ranked ligand conformations to the query, with a higher score assigned to a ligand conformation with a higher rank. We used *k* = 20 in this study. The second term is to normalize the score by the number of conformations from ligand *L* included in the database. The ligand with the highest *PocketScore* is predicted to bind to the query pocket.

#### 3.6.2. PDBbind Dataset

The PDBbind [38,39] “core set” provides 210 protein-ligand complexes non-redundantly sampled from 1300 protein–ligand complexes [38]. It covers 70 different proteins, each of which contains three protein–ligand complexes with different binding affinities, which makes it ideal for optimizing the search algorithm of PL-PatchSurfer. All the protein–ligand complexes in the PDBbind core set were pre-processed with added hydrogen atoms and were therefore used directly without additional preparations.

**Figure 8:**
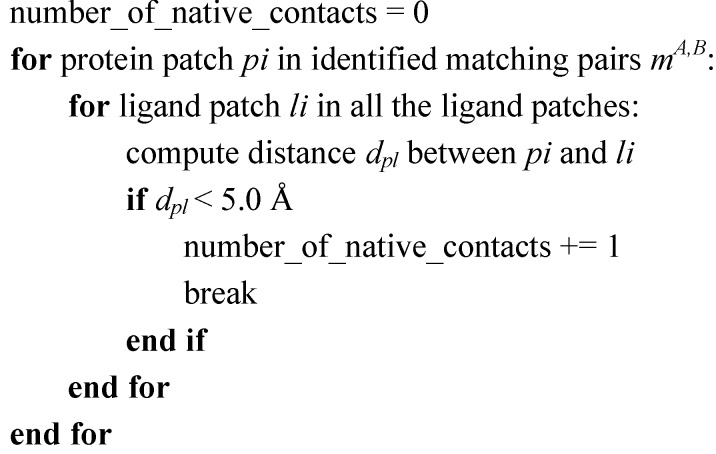
Pseudo-code for computing the maximum number of native contacts.

To optimize PL-PatchSurfer’s performance in identifying the matching patches that reproduce the protein–ligand interactions, we computed the match success rate for each protein–ligand complex structures in the PDBbind core set. For each protein–ligand complex, we identified the protein patches and the ligand patches (Figure 7). We say a protein patch and a ligand patch form a “native contact” if the distance between their patch centers are within a cutoff distance, either 3.0, 4.0, 5.0 or 6.0 Å. The 5.0 Å distance cutoff is frequently-used empirical distance cutoff for computing the steric interactions between a ligand and a protein in many scoring functions [56,57]. We then computed the maximum number of correct contacts can be formed between the protein and the ligand for each complex structure. The pseudo-code to compute such maximum number of “native contacts” is provided in Figure 8.

Matching patch pairs will be identified by PL-PatchSurfer. If the distance between the centers of the identified matching patch pair is within a cutoff distance in the crystal structure, we count it as a success match. The match success rate for each protein-ligand complex structure is then defined as the number of success match identified by PL-PatchSurfer divided by the maximum number of correct contacts can be formed for the protein–ligand complex. The overall success rate is then computed by averaging the success rate over all the protein–ligand structures.

An optimization program was constructed to search for the best coefficients in Equation (4) that will lead to the largest average success rate. First we reduced the three parameters *w*_1_, *w*_2_, and *w*_3_ in Equation (4) into two parameters *a*, *b* by

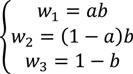
(17)


Therefore *w*_1_ + *w*_2_ + *w*_3_ = 1. The rationale is to reduce the degree of freedom in searching for the optimal parameters therefore increase the searching speed. During the process of searching for the optimal parameter set, *a* and *b* were allowed to change from 0.0 to 1.0 with a step size of 0.1. The parameter set that leads to the maximum success rate was taken as the final optimized weights in Equation (4).

#### 3.6.3. DUD Set

The dictionary of useful decoys (DUD) [50] dataset was used to perform virtual screening studies. The DUD dataset contains 40 protein targets and a set of active and decoy ligands corresponding to each target. There are 2950 active ligands in total, each of which has 36 physically similar but topologically different decoy ligands. In the current version of PL-PatchSurfer, the parameters of ions and cofactors were not included. Therefore, the four metalloenzymes, two folate enzymes, and five other enzymes (aldose reductase, enoyl ACP reductase, glycogen phosphorylase β, purine nucleoside phosphorylase, and *S*-adenosyl-homocysteine hydrolase) were excluded in our virtual screening experiment. The human shock protein 90 and thymidine kinase were excluded due to the failure of APBS in processing the protein structure caused by the missing atoms. Hydroxymethylglutaryl-CoA reductase and trypsin were excluded due to the failure of APBS in processing most of the active and decoy ligands due to incompatible atom typing to APBS. For cyclooxygenase-2 (COX2) and epidermal growth factor receptor (egfr), over 10,000 decoys are present in the DUD set. To speed up the testing process, a subset of 30 actives and 1080 decoys was randomly selected.

For each ligand in the DUD dataset, we generated a maximum of 20 ligand conformations using OpenEye Omega. The surface patches for each ligand conformation were then identified using PL-PatchSurfer and stored in the DUD library. The surface patches were also calculated for the protein. The pocket patches were then used to search against the DUD ligand library of each target class. The fit of each ligand conformation into the protein pocket was measured by *Totalscore_PL_* (Equation (8)). The final score for each ligand was calculated by averaging the scores of its top-10 best fitted ligand conformations. The ligands for each protein system were ranked based on their final score. The Receiver Operating Characteristic (ROC) curve displaying the fraction of ranked actives (true positive rate) at a given fraction of ranked decoys (false positive rate) was plotted for each run. The area-under-the-curve (AUC) was calculated for each ROC curve and used to assess the overall enrichment quality.

To optimize the weights in Equation (8), we randomly selected 12 of the DUD set to form the training set and left the other 13 targets as the testing set. During the optimization process, the weight *w* in Equation (8) was allowed to change from 0.0 to 1.0 with an interval of 0.1. The AUC value for each protein target in the training set was calculated for each weight. The weights, *w*_1_ = 0.8, *w*_2_ = 0.0, *w*_3_ = 0.1, that provide the optimal average AUC of all the training proteins were taken as the best parameters. To test the generalization of the trained parameters, we performed a two-fold cross-validation by switching the training and testing set. The resulting weights are *w*_1_ = 1.0, *w*_2_ = 0.0, *w*_3_ = 0.6.

The target-specific optimizations were performed for AChE, PPAR, COX-2, PDGFrb, and thrombin. For each target, 10 active and 360 decoy ligands were randomly selected from the ligand dataset to form the training set. The remaining ligands for each target were left as the testing set. Each weight in *Totalscore_PL_* (Equation (8)) was changed from 0.0 to 1.0 with an interval of 0.1 to identify the set of weights that can achieve the optimal AUC value on the training set for each target. The optimized weights were then tested on the testing set of each target.

## 4. Conclusions

We have developed a new patch-based ligand analysis program, PL-PatchSurfer. First we have demonstrated that PL-PatchSurfer works well in predicting binding ligands for pockets of target proteins. By identifying compatible patch pairs from a target pocket and candidate ligands, PL-PatchSurfer showed higher success rate than Patch-Surfer even when the native conformation of ligands were excluded and also better or comparable results than PatchSurfer when Omega-generated ligand conformations were used. Thus, PL-PatchSurfer is a promising new method for binding ligand prediction that can give a clue for biological function for protein structures of unknown function. We then optimized the search algorithm of PL-PatchSurfer using the PDBbind data set to improve its success rate in identifying native contacts. Finally, we explored the possibility of PL-PatchSurfer in virtual screening experiment. Performance comparison against PharmDock, which was shown to perform better or comparable to other existing methods, showed that PL-PatchSurfer has better in early enrichment of actives than PharmDock. Detailed analyses showed that PL-PatchSurfer detected patches corresponding patches in pockets and ligands in correct places.

Comparing to existing atom-based docking programs, PL-PatchSurfer also has a great advantage in its computational efficiency. PL-PatchSurfer, on average, needs 0.7 s to search one ligand against a given target on a single core of an Intel i7-3820 computer. This is in contrast to the more time consuming protein–ligand docking programs, which normally need 30 to 250-fold longer time to complete the docking of one ligand (Table 4). This speed improvement can result in substantial difference in computational time, in the order of days and weeks, considering practical virtual screening situations where millions of compounds are matched to a target.

So when PL-PatchSurfer can be most useful in practice? Obviously, PL-PatchSurfer should be very useful in function prediction of proteins of unknown function as it was shown to be better than PatchSurfer that was already better than many existing methods. Moreover, PL-PatchSurfer will also be effective in virtual screening as a complementary tool to existing docking-based methods. It has been discussed that current docking-based virtual screening methods still have limitations in identifying actives [24]. Because PL-PatchSurfer employs a completely different approach of surface patch matching yet performs competitively with existing methods, we believe that this surface-patch approach has potential to significantly enhance the design of new ligands in several challenging drug–target areas including G-protein coupled receptors, fragment-based drug design and protein–protein interactions (PPI). In addition, we plan to investigate the use of PL-PatchSurfer to assess ligandability—The relative ability of a protein target to productively interact with a drug-like ligand—In a collection of known PPI systems.
